# Verbal worry facilitates attention to threat in high-worriers^☆^

**DOI:** 10.1016/j.jbtep.2013.05.006

**Published:** 2014-03

**Authors:** Marc O. Williams, Andrew Mathews, Colette R. Hirsch

**Affiliations:** a King's College London, Institute of Psychiatry, UK; b University of California, Davis, USA

**Keywords:** Worry, Attention, Imagery, Verbal-linguistic processing, Dot probe

## Abstract

**Background and objectives:**

Worry is predominantly a verbal-linguistic process with relatively little imagery. This study investigated whether the verbal nature of worry contributes to the maintenance of worry by enhancing attention to threat. It was hypothesised that verbal worry would lead to greater attentional bias to threat than imagery-based worry.

**Methods:**

Fifty high-worriers were randomly assigned to one of two groups, one in which they were instructed to worry in a verbal way and one in which they worried in an imagery-based way, before completing a dot probe task as a measure of attention to threat-related words.

**Results:**

Those who worried in verbal form demonstrated greater attentional bias to threat than did those who worried in imagery-based form. These findings could not be accounted for by group differences in personal relevance of or distress associated with worry topics, state mood following worry, levels of the relatedness of participants' worries to stimuli on the dot probe task, trait anxiety, general propensity to worry, nor adherence to the worry training.

**Limitations:**

The present study only included word stimuli in the dot probe task; inclusion of images would allow for firmly rejecting the hypothesis that the attention effects observed following verbal worry were merely a result of priming verbal threat representations. Also, future studies could include a further control group that does not engage in any form of worry to ascertain that verbal worry increased attentional bias rather than imagery decreasing pre-existing attentional bias.

**Conclusions:**

Possible mechanisms underlying this effect of verbal worry on attention to threat are discussed, together with clinical implications of the current findings.

## Highlights

• Worry is predominantly a verbal-linguistic process with relatively little imagery.
• High-worriers worried in verbal or imagery form before an emotional dot probe task.
• Verbal worry is associated with attentional bias for threat words.
• Imagery-based worry is not associated with an attentional bias for threat words.
• The verbal nature of worry has a causal role in its maintenance.

## 1 Introduction

Worry is a cardinal feature of generalised anxiety disorder (GAD). Why some people continue to worry excessively when it appears to maintain anxiety with little objective benefit remains an unanswered question. Worry is known to be characterised by verbal-linguistic processing, which becomes more dominant over imagery-based processing as people move from thinking in a relaxed fashion to worrying (Borkovec & Inz, 1990). Borkovec, Alcaine, and Behar (2004) hypothesised that verbal worry might be negatively reinforced as it suppresses aversive mental imagery and associated somatic symptoms of anxiety but that, in doing so, it interferes with the prolonged activation of the relevant “fear structure” stored in memory that is required for habituation and corrective learning about the feared topic (as per Foa and Kozak's (1986) emotional processing theory). This “fear structure” thereby remains unprocessed and, as a result, continues to be activated. In support of this hypothesis, Butler, Wells, and Dewick (1995) showed that participants who had been shown an anxiety-provoking video and who were then instructed to worry about it in a verbal way experienced a greater decrement in anxiety than those who were instructed to generate mental images from the video. However, those who worried in a verbal way reported more frequent intrusions relating to the video they had seen in the days that followed, compared with those who generated images about the video.

Attention is another process that could be affected by the verbal-linguistic nature of worry. Anxiety, which is a major affective component of worry (Andrews & Borkovec, 1988), is known to be associated with attentional bias to threats. MacLeod, Mathews, and Tata (1986) conducted a landmark study into biased attention in people with GAD using a dot probe task. Participants' reaction times (RTs) were quicker when responding to dots replacing threat words than to dots replacing non-threat words, taken by the authors to indicate preferential attention to threats relative to neutral words and implying a similar bias to threatening information encountered in daily life.

Researchers have begun to explore the link between worry and attention more directly. For example, Krebs, Hirsch, and Mathews (2010) manipulated attention to threat cues in people without excessive worry using a training task developed by MacLeod, Rutherford, Campbell, Ebsworthy, and Holker (2002) and showed that inducing attentional bias to threat gave rise to more negative thought intrusions on a breathing focus task than facilitating an equivalent bias to neutral stimuli. This suggests that habitual attention to threat has a role in maintaining worry.

To our knowledge, only one study (Oathes, Squillante, Ray, & Nitschke, 2010) has investigated the reverse direction of influence, i.e., whether worry can lead to changes in attention to threats. Oathes et al. (2010) allocated participants scoring in the “low normal” worry range (Penn State Worry Questionnaire scores between 20 and 50; PSWQ; Meyer, Miller, Metzger, & Borkovec, 1990) to one of two experimental manipulations, in one of which participants were instructed to worry and another in which they performed an arithmetic task (the control condition). Both groups then completed a dot probe task to assess attention. Participants saw some word pairs consisting of one threat and one non-threat word (valenced trials) and others of two non-threat words (non-valenced trials), followed by a target (dot) in the location of one of the words. Participants were required to pay attention to the word appearing in the upper location. The authors found that, within the worry condition only, responses were quicker to probes appearing in the attended top location on valenced trials than on non-valenced trials. While this is an interesting finding, it does not constitute attentional bias to threat and is not a calculation found in previous research. The authors did not find evidence for biased attention to threat following worry using the traditional measure of MacLeod et al. (1986), i.e., speeded responses to probes in the prior location of threat compared with non-threat words.

The present study was designed to provide a further test of the prediction that worry can augment attention to threat cues. Rather than testing low-worriers (as did Oathes et al., 2010), who often tend to avoid threat cues, we studied non-clinical high-worriers, a group we thought more likely to reveal any effect that worry might have on attentional bias (Hirsch & Mathews, 2012). Furthermore, instead of using an unrelated arithmetic task as a comparison condition, we chose to contrast two different forms of worry, i.e., verbal or mental imagery of the same negative content. As well as providing a comparison condition better matched for exposure to worry content per se, this allowed us to address a specific hypothesis about the mechanism by which worry might facilitate attention to threat, i.e., that the verbal-linguistic nature of worry has a causal role in this regard. More specifically, we propose that verbal-linguistic worry could facilitate attentional bias to threat via the aforementioned mechanism proposed by Borkovec et al. (2004), in which verbal thought interferes with the processing of “fear structures”, whereas we would expect imagery-based worry to lead to fuller emotional processing and therefore less attentional bias to threat.

In the present study high-worriers were randomly allocated to one of two groups, one in which the instruction was to worry in the usual verbal manner and another in which the instruction was to worry in an imagery-based way. It was predicted that subsequent attention to threat would be more evident after verbal worry than when people imagined worry-related outcomes.

## 2 Method

### 2.1 Participants

Sixty high-worriers who spoke English as a first language attended the experimental session. They were recruited using an advertisement on a website and scored 56 or above^1^ on the PSWQ at screening. Ten people who attended the experimental session were excluded from the study at various stages (see Section 2.4 for a summary of the different stages). Five people were excluded due to no longer scoring 56 or above on the PSWQ on attending the experimental session, and two people allocated to the Imagery group chose to discontinue the study during the worry training.^2^ Participants were also required to meet two further rating criteria in each 2 min worry period in the worry phase and worry reactivation phase: one criterion required at least 60% of thought content to be negative in valence, and the other required at least 60% of thought content to be in the designated mentation style (i.e., verbal or imagery, depending on group allocation) and/or at most 40% of thought content to be in the non-designated style.^3^ Three people were excluded from the study for not reaching one of these criteria during one of the 2 min worry periods in the worry phase and worry reactivation phase, two in the Verbal group and one in the Imagery group.

There were 25 participants in each group in the final sample. No significant difference was found in the number of females in the Verbal and Imagery groups, 20 vs. 21, Fisher's Exact Test *p* = 1. The Verbal and Imagery groups did not differ in age, *Mean* = 26.68, SD = 8.70 vs. *Mean* = 26.08, SD = 8.73, Mann Whitney's *U* Test *p* = .86. As shown in Table 1, the two groups did not differ in their scores on the PSWQ, the trait scale of the State-Trait Anxiety Inventory (STAI-T; Spielberger, Gorsuch, Lushene, Vagg, & Jacobs, 1983), or the Worry Domains Questionnaire-Short Form (WDQ-SF; Stöber & Joormann, 2001).

### 2.2 Self-report questionnaires and ratings

#### 2.2.1 Penn state worry questionnaire

The PSWQ (Meyer et al., 1990) comprises 16 statements relating to worry, which participants rate from 1 (not at all typical of me) to 5 (very typical of me). Studies report the PSWQ to have high short-term retest reliability and convergent and criterion related validity (Brown, Antony, & Barlow, 1992; Davey, 1993). Molina and Borkovec (1994) showed the PSWQ to have high internal consistency (*α* = .91).

#### 2.2.2 Trait version of the state-trait anxiety inventory

The STAI-T (Spielberger et al., 1983) consists of 20 statements relating to anxiety, which participants rate from 1 (almost never) to 4 (almost always). The STAI-T has demonstrated good convergent validity (Peterson & Reiss, 1987), concurrent validity (Spielberger, Ritterband, Sydeman, Reheiser, & Unger, 1995), construct validity (Smeets, Merckelbach, & Griez, 1997), and test–retest reliability (Rule & Traver, 1983). Spielberger et al. (1983) reported high internal consistency for the STAI-T (*α* = .90).

#### 2.2.3 The worry domains questionnaire short form

The WDQ-SF (Stöber & Joormann, 2001) is a measure of predominant domains of worry, consisting of 10 items, based on the original Worry Domains Questionnaire (WDQ) of Tallis, Eysenck, and Mathews (1992). Participants rate how much they worry about each item, ranging from 0 (not at all) to 4 (extremely). Davey (1993) showed the WDQ to have high internal consistency (*α* = .91).

#### 2.2.4 Mood rating scales

Three visual analogue Mood Rating Scales were completed by participants at various points during the study. Anxiety, depression and happiness were rated on a 10 cm line with anchors of “not at all” and “extremely”.

### 2.3 Experimental tasks

#### 2.3.1 Worry training

Participants in the Verbal group were instructed to worry in a verbal way, and those in the Imagery group to worry using imagery. Training for participants in the Verbal group began with their being asked to think in words, sentences and questions about the topic of “friendship”. Then this group went on to think in words, sentences and questions about the negative aspects of four scenarios: the first concerning a financial worry (1 min); the second concerning a social worry (1 ½ minutes); the third concerning a physical health worry (2 min); and the fourth concerning a relationship worry (2 min). Training for the Imagery group also began with thinking about the topic of “friendship”, but as a mental image. This group then went on to think about the same four scenarios except that, prior to thinking about each one, they were instructed to consider specific feared outcomes. These participants then proceeded to imagine themselves in each of the scenarios in a time- and location-specific manner, “as though it were happening now”, focussing on the negative aspects.

After each scenario, participants completed ratings to indicate the degree to which they had been thinking in their designated and non-designated mentation style, and the extent of their negative/neutral/positive thoughts. Where necessary, participants were provided with feedback to help them to adhere to their designated mentation style and to focus on the negative content of the scenario. For example, a participant in the Verbal group might have been advised to move away from persistent mental imagery by describing mental images to themselves using words and then forming questions relating to those words, which could then lead on to mental running commentary. A participant in the Imagery group might have been instructed to make a vaguely formed mental image more vivid by “tuning into each of the five senses, including what you can hear, smell, and feel in the image”.

#### 2.3.2 Worry phase

Participants identified a currently concerning worry topic. Worry topics were then rated by participants using three 10 cm lines to measure how “Personally Relevant” and “Distressing” they were, with anchors of “not at all” and “totally”. Each participant then briefly discussed the worry with the experimenter in order to activate the worry in the participant's mind and to ensure that it was connected with a negative future event. Participants were then asked to worry for three periods of 2 min each in their designated mentation style, as they had learnt to do in the worry training. The experimenter left the room for each 2 min period and, prior to leaving, he reminded the participant to worry in their designated style. After each 2 min worry period, participants rated the proportion of their thoughts that had been positive, neutral and negative during the worry period and the extent to which they had worried in their designated and non-designated mentation style during the worry period, using 10 cm lines with anchors of “not at all” and “totally”.

#### 2.3.3 Dot probe task

Threat words were chosen to fit the domains of worry identified by Tallis et al. (1992) in the development of the WDQ, whose cluster analysis revealed six domains of worry in the general population: relationships, lack of confidence, aimless future, work incompetence, financial, and socio-political, each containing five subdomains. Two further domains were added: physical and social, as these are common worry topics in pathological worriers. This gave rise to a total of eight domains, each with five subdomains (40 subdomains in total). One word was chosen to represent each of the 40 subdomains, which became the 40 threat words (see Table 2) used in the current study's dot probe task. These words were first piloted by presenting them to seven high-worriers. For each word, pilot participants were instructed to “indicate how negative/positive you find the word”, using a 7-point scale ranging from −3 (extremely negative) to 0 (neutral) to 3 (extremely positive). Only those threat words with a mean rating of −1 or below were included in the final corpus of threat words, and when two words represented the same worry domain, the word with the most negative rating was chosen. Non-threat words were also piloted using the same method. Only words that were given a rating of 0 or above were included in the final corpus of non-threat words. Threat and non-threat words were paired to give rise to two types of trial on the dot probe task: valenced trials (threat/non-threat word pairs) and non-valenced trials (non-threat/non-threat word pairs). Forty valenced and 40 non-valenced word pairs were developed.

Each word pair contained two words of equal length. Within the valenced word corpus, the average standard frequency index (SFI) of threat words, *M* = 40.91, SD = 12.04, was matched with that of the non-threat words, *M* = 40.94, SD = 5.07. The average SFI of valenced word pairs, *M* = 40.93, SD = 9.18, was matched with that of non-valenced word pairs, *M* = 40.43, SD = 2.11. The average length of valenced word pairs, *M* = 7.23, SD = 2.06, was matched with that of non-valenced word pairs, *M* = 7.47, SD = 2.11.

In the dot probe task (based on MacLeod et al., 1986), trials involved a fixation cross presented for 1000 ms in the middle of the screen, followed by a word pair in which the words were presented one above the other, appearing above and below the fixation cross. Word pairs were presented for 200 ms before being replaced by a probe (“.” or “..”) in the location of either the top or the bottom word. Participants were required to press a key (either “c” or “m” on the keyboard, which were labelled with “.” and “..”, respectively), to match the probe they saw on the screen. Participants were instructed to respond to the probe as quickly as possible without making mistakes.

Each word in the valenced word pairs was presented twice in the top location and twice in the bottom location and, for each location, the word was followed by a probe in its location once and a probe in the location of the other word once. This gave rise to four different conditions for valenced trials, which can be summarised as: threat-top/probe-top; threat-top/probe-bottom; threat-bottom/probe-top; threat-bottom/probe-bottom. For non-valenced trials, each word in a pair was shown once in each of the following conditions: word-top/probe-top; word-top/probe-bottom; word-bottom/probe-top; word-bottom/probe-bottom.

Participants first completed a practice task, consisting of five trials in which word pairs referred to household objects. The main task comprised two blocks, each containing 20 valenced word pairs and 20 non-valenced word pairs. Each pair was repeated (in random order) across the four conditions, giving rise to 160 trials in each block (80 valenced and 80 non-valenced), and 320 trials in total. In between the two blocks there was a worry reactivation phase (see Section 2.3.4).

#### 2.3.4 Worry reactivation phase

After the first block of the attention task, participants were asked again to worry about the same worry topic that they had worried about previously, this time for another 2 min in their designated mentation style. After worry reactivation, participants rated the proportion of their thoughts that had been positive, neutral and negative and the extent to which they had worried in their designated worry style, or in their non-designated worry style, during the worry period, using 10 cm lines with anchors of “not at all” and “totally”.

#### 2.3.5 Word rating task

Participants were presented with the 40 threat words that had appeared in the dot probe task, shown individually on a computer screen and were asked to rate how related each word was to what they had been worrying about during the worry phases, using a 4-point scale of “not at all related”, “slightly related”, “moderately related”, and “extremely related”.

### 2.4 Procedure

After random assignment to one of the two groups (Verbal or Imagery) participants completed the PSWQ, STAI-T, and WDQ, and rated their baseline state mood on the Mood Rating Scales. Next, they completed the dot probe practice task, followed by the worry training and the worry phase. Following this, participants completed the first block of the dot probe task. After the worry reactivation phase, participants completed the second block of the dot probe task, followed immediately by the word rating task. Mood and worry ratings were completed after the dot probe practice task, the worry training and worry phase, and after the worry reactivation phase.

## 3 Results

### 3.1 Dot probe task

#### 3.1.1 Accuracy

Accurate responses to targets averaged 98.11% (SD = 6.98), with no significant difference in accuracy between the Verbal and Imagery groups, *M* = 313.40, SD = 8.06 vs. *M* = 314.52, SD = 5.82, *t*(48) = .56, *p* = .58, *d* = .16. Therefore, any group differences in latencies could not be attributed to differential speed-accuracy trade-offs.

#### 3.1.2 Response latencies

For ease of interpretation, latency data for valenced trials (threat – non-threat) were analyzed by collapsing conditions threat-top/probe-bottom and threat-bottom/probe-top to make “RT to probes at neutral location” and collapsing conditions threat-top/probe-top and threat-bottom/probe-bottom to make “RT to probes at threat location”. An Attentional Bias Index (ABI) was then computed separately for the Verbal and Imagery groups: (mean [RT for probes at neutral location] – [RT for probes at threat location]). To minimise the influence of outlying data, median RTs were computed for each condition of valenced trials^4^ before these were collapsed to calculate the ABI scores. Median RTs for valenced and nonvalenced trials can be found in Table 3.

Two one-sample *t*-tests compared ABI scores of the two groups with zero to estimate the extent of biased attention. The ABI score for the Verbal group was significantly larger than zero, *M* = 7.39, SD = 17.87, *t*(24) = 2.07, *p* = .05, *d* = .41, reflecting speeded responses to threat words relative to neutral words, whereas the ABI score of the Imagery group did not significantly differ from zero, *M* = −2.69, SD = 13.17, *t*(24) = −1.02, *p* = .32, *d* = .20. An independent samples *t*-test was also conducted, which showed the ABI score of the Verbal group to be significantly larger than the ABI score of the Imagery group, *M* = 7.39, SD = 17.87 vs. *M* = −2.69, SD = 13.17, *t*(48) = 2.27, *p* = .03, *d* = .98. This reflected significantly more speeded responses to threat words in the Verbal group relative to the Imagery group.

### 3.2 Mood ratings

Two mixed-model ANOVAs were conducted to examine the effects of the experimental manipulations on anxiety and depression, with a repeated measures factor of Time (Pre-Worry; Post-Worry; Post-Worry Reactivation), and a between subjects factor of Group (Verbal; Imagery). For both ratings, there was a main effect of Time (anxiety *F*(2,96) = 22.63, *p* < .001, *η*^2^ = .32; depression *F*(2,96) = 17.43, *p* < .001, *η*^2^ = .27). Further *t-*tests showed that the main effect of Time reflected significant increases in both anxiety and depression from before the worry phase to afterwards (*M* = 5.79, SD = 2.24 vs. *M* = 7.17, SD = 1.97, *t*(49) = 5.53, *p* < .001, *d* = 1.58, and *M* = 4.22, SD = 2.57 vs. *M* = 5.45, SD = 2.84, *t*(49) = 5.17, *p* < .001, *d* = 1.48), but no significant decrease from after the worry phase to after the worry reactivation phase (*M* = 7.18, SD = 1.97 vs. *M* = 7.04, SD = 1.70, *t*(49) = .87, *p* = .39, *d* = .25, and *M* = 5.45, SD = 2.84 vs. *M* = 5.18, SD = 2.66, *t*(49) = 1.73, *p* = .09, *d* = .49), suggesting that the worry reactivation phase was sufficient to maintain the effects of worry. No effects involving Group approached significance, indicating that worrying in verbal compared with imagery-based form did not have differential effects on state mood.

### 3.3 Worry topic ratings

Two paired-samples *t*-tests were conducted to examine the extent of distress and personal relevance associated with the worries chosen by the two groups. There was no significant difference in the rated personal relevance of worries chosen by the Verbal and Imagery groups, *M* = 8.78, SD = 1.25 vs. *M* = 8.60, SD = 1.35, *t*(48) = .48, *p* = .69, *d* = .14, nor for rated distress associated with worries chosen by the Verbal and Imagery groups, *M* = 7.96, SD = 1.57 vs. *M* = 7.60, SD = 1.65, *t*(48) = .79, *p* = .78, *d* = .22.

### 3.4 Mentation ratings

#### 3.4.1 Valence

A 3 × 2 mixed-model ANOVA was conducted (Valence × Group) to compare the Verbal and Imagery groups on percentage of negative, positive, and neutral thoughts during the 2 min worry periods (scores were averaged over the three 2 min periods in the worry phase and the 2 min period in the worry reactivation phase). There was a main effect of Valence (Negative *Mean* = 90.17, SD = 9.07; Positive *Mean* = 1.78, SD = 3.51; Neutral *Mean* = 7.60, SD = 7.95, *F*(2, 96), *p* < .001, *η*^2^ = .99), confirming that participants were experiencing mostly negative thoughts, as instructed. There was no interaction effect of Valence × Group, *F*(2, 96) = .88, *p* = .42, *η*^2^ = .02, indicating that the two groups did not differ with regard to the percentage of positive, negative and neutral thought content during the worry periods overall. Further analysis involving time of rating as a factor did not reveal an interaction of Time × Valence × Group, indicating that the two groups did not differ in how Valence varied over time, *F*(6, 288) = .65, *p* = .69, *η*^2^ = .01.

#### 3.4.2 Mentation style

An independent samples *t*-test was conducted to compare the Verbal and Imagery groups on the percentage of their thoughts that were in the designated mentation style (scores were averaged over the three 2 min periods in the worry phase and the 2 min period in the worry reactivation phase). No significant group difference was found in the percentage of thoughts in the designated mentation style in the Verbal and Imagery groups, *M* = 90.39, SD = 8.35 vs. 85.40, SD = 11.45, *t*(48) = 1.76, *p* = .09, *d* = .50.

### 3.5 Word ratings

To check for any differences in perceived relevance of the worry-related words used, the number of words rated as highly relevant by participants in each group was analysed. Inspection of distributions revealed an extreme outlier within the Verbal group, so the groups were compared using Mann–Whitney’s *U* Test. This showed no significant difference in the mean number of words rated by participants within groups as highly relevant to their worry (*Z* = −.48, *p* = .63).

## 4 Discussion

To our knowledge this is the first study to demonstrate that the verbal-linguistic processing style usually adopted during worry is associated with greater attention to threat. High-worriers who worried in the usual verbal manner showed evidence of selective attention to threat, whereas those who worried in imagery-based form did not. This contrasted with the results reported by Oathes et al. (2010), which could be because Oathes et al. sampled “low-normal” worriers, who have sometimes been found to avoid threat cues.

The present findings are consistent with the hypothesis that typical worry augments attention to threat cues. The results cannot easily be explained by group differences in trait anxiety, propensity to worry, personal relevance of and distress associated with worry topics, nor the number of words in the dot probe task that were highly relevant to participants' worry topics, since the groups did not differ on any of these measures. Nor did the two groups differ in their adherence to the worry training, i.e., there was no difference in adherence to the designated mentation style and percentage of thoughts that were positive, neutral and negative over the 2 min worry periods.

Of particular note is the fact that the two groups did not differ in anxious or depressed mood following the worry phase and the worry reactivation phase. It was mentioned in the introduction that one reason to expect group differences in attentional bias to threat is a mechanism proposed by Borkovec et al. (2004), in which verbal worry might interfere with habituation and corrective learning about the “fear structure” by suppressing aversive imagery and its associated anxiety. However, the data of the current study are not fully supportive of this explanation for the results, as one would expect the Verbal group to report lower anxiety following worry than the Imagery group if verbal worry had suppressed anxiety.

At first glance it may seem surprising that imagery did not elicit a greater emotional response than verbal worry. While there is indeed evidence that imagery is typically associated with greater emotional response than is verbal representation of *novel* events (e.g., Holmes & Mathews, 2010), the present finding is consistent with other studies that have found that worrying about typical concerns in verbal vs. imagery-based form does not differentially affect state mood (e.g., Behar, Zuellig, & Borkovec, 2005; Leigh & Hirsch, 2011; Stokes & Hirsch, 2010). One reason for this is that participants were required to focus on their current main worry topic, which, by definition, had been thought about repeatedly prior to attending the experimental session, leading to some degree of emotional habituation prior to worrying during the experiment in either verbal or imagery form. Another possibility is that retrospective ratings of mood are not sensitive to subtle mood changes.

What other mechanisms could account for the group difference in attentional bias to threat? Holmes and Mathews (2010) proposed two notable mechanisms that deserve consideration, namely that negative mental imagery can be rescripted into a more benign form, and that the individual exposed to imagery might come to appreciate the difference between imagery and immediate, real-world perceived stimuli. However, the lack of a group difference in state mood following worry makes both of these proposals unlikely accounts for the current study's findings.

Another possibility is that the group difference in attention to threat arose from a more general cognitive impairment following verbal worry. Leigh and Hirsch (2011) found high-worriers to have less available working memory capacity during verbal than during imagery-based worry, so that it might be argued that the ability to voluntarily control attention to threat was depleted in the present group engaging in verbal worry, thus revealing an apparently greater attentional bias. We think this alternative possibility is unlikely as it would also lead us to expect generally impaired performance in the Verbal group, whereas the main effect of Group on accuracy and overall latencies on the dot probe task did not approach significance.

Butler (1994) noted that typical (verbal) worry involves consequences that are expressed in abstract as opposed to concrete terms, such as “what if I get in a muddle?” or “something dreadful might happen–or have happened” (p. 223). Indeed, participants in the verbal group of the current study were instructed merely to think in words, sentences and questions about their worry during the worry phase and the worry reactivation phase, with no references made to specific feared outcomes, whereas the specific feared outcomes of those in the Imagery group were discussed prior to the worry phase, and participants were instructed to situate their mental images concretely in space and time. As such, it is likely that participants in the Verbal group engaged in more abstract processing than those in the Imagery group, which might have led to attentional bias that operated on a more abstract level, i.e., toward threat words encompassing diverse themes. Future studies could include a measure to test out the causal effect that concreteness of worry might have on attentional bias to threat by asking participants to rate their worry content after each worry period on a continuum between abstract and concrete (see Stöber, Tepperwien, & Staak, 2000).

The present study has two main limitations that should be borne in mind. First, it is possible that the attention effects observed, in which RTs to threat words were speeded in the Verbal compared with the Imagery group, depended on priming only verbal representations of threat in the Verbal group, and that imagery might have similarly primed attention to perceptual representations of threat, but that such an effect would not have been detected in the present dot probe task that used only words. We think this is unlikely to account for the results because, while imagery-based worry might be expected to prime some specific mental images, as argued in the previous paragraph, this would be unlikely to prime a range of stimuli encompassing many themes as might be the case in verbal worry. Nonetheless, this should be tested using pictures as well as words in the dot probe task following different forms of worry.

Second, because we did not assess attention to threat before the worry phase (to avoid practice and fatigue effects, as well as habituation to the worry-related words), we cannot be certain that imagery did not *decrease* pre-existing attentional bias, rather than verbal worry increasing it. There seems no obvious reason to expect such an effect, but future research might address this by including a control group who are given a distractor task to subdue worry, in order to estimate baseline attentional bias.

It would be premature to draw conclusions about clinical conditions such as GAD on the basis of results from the current study, which did not involve a clinical population. However, if future studies were to replicate the current findings using a clinical sample then this could have important implications for the incorporation of imagery-based techniques in treating pathological worry.

In summary, the current study demonstrates that worrying in verbal form results in more attention being allocated to threat cues than is the case following imagery-based worry. This finding adds to previous findings that verbal worry increases subsequent negative thought intrusions and provides further evidence that the verbal nature of worry is implicated in the persistence of worry. Further research with clinical groups is indicated to determine whether the findings can be replicated in pathological worriers, and to explore the implications for imagery-based interventions in therapy.

## Figures and Tables

**Table 1:** Mean questionnaire scores (standard deviations in parentheses).

	Verbal	Imagery	Test	*t*(48)	*p*	*η*^2^
PSWQ^a^	65.68 (6.52)	64.68 (5.40)	Independent samples *t*-test	.60	.56	.17
STAI-T^b^	57.36 (7.81)	55.52 (8.48)	Independent samples *t*-test	.80	.43	.23
WDQ-SF^c^	26.00 (6.86)	27.52 (5.46)	Independent samples *t*-test	.87	.39	.25

a PSWQ = Penn State Worry Questionnaire.

b STAI-T = Trait scale of the State-Trait Anxiety Inventory.

c WDQ-SF = Worry Domains Questionnaire-Short Form.

**Table 2:** Threat words included in the attention probe task, and their associated domains of worry.

Threat word	Domain
Lonely	Relationships
Ugly	Relationships
Shunned	Relationships
Breakup	Relationships
Unloved	Relationships
Coward	Lack of confidence
Criticised	Lack of confidence
Wimp	Lack of confidence
Stupid	Lack of confidence
Insecure	Lack of confidence
Useless	Aimless future
Failure	Aimless future
Unemployed	Aimless future
Aimless	Aimless future
Absentminded	Aimless future
Late	Work incompetence
Incapable	Work incompetence
Incompetence	Work incompetence
Lazy	Work incompetence
Deadlines	Work incompetence
Bankrupt	Financial
Hardship	Financial
Debt	Financial
Poverty	Financial
Bills	Financial
Starvation	Socio-political
Abuse	Socio-political
Landfill	Socio-political
Torture	Socio-political
Cruelty	Socio-political
Agony	Physical
Cancer	Physical
Choking	Physical
Crippled	Physical
Assault	Physical
Worthless	Social
Inferior	Social
Boring	Social
Humiliated	Social
Despised	Social

**Table 3:** Median reaction times for all conditions in valenced trials and nonvalenced trials (standard deviations in parentheses).

	Threat position	Probe position	
		Top	Bottom
*Valenced trials*			
Verbal	Top	508.68 (67.36)	538.70 (75.05)
	Bottom	517.00 (73.86)	532.24 (70.15)
Imagery	Top	488.92 (45.34)	524.62 (51.08)
	Bottom	486.50 (44.66)	527.58 (54.14)
*Nonvalenced trials*			
Verbal		509.38 (71.07)	538.36 (69.16)
Imagery		484.82 (42.69)	519.40 (47.96)

## Footnotes

1 Prior research (Molina & Borkovec, 1994) has found a PSWQ score of 56 to fall one standard deviation below the mean of individuals diagnosed with GAD.

2 These participants had found the worry training to be distressing.

3 These criteria of valence and mentation style were chosen on the basis of previous studies, which used similar but slightly less stringent criteria (e.g., Leigh & Hirsch, 2011; Stokes & Hirsch, 2010).

4 Although Q–Q plots of the four conditions resulting from four combinations of threat location and probe location (threat top/probe top; threat top/probe bottom; threat bottom/probe top; threat bottom/probe bottom) were found to be approximately normal, two extreme outliers were found in the box plots, both in the Imagery group. A logarithmic transformation was conducted but these outliers remained, therefore ABI scores were calculated with extreme outliers included. When ABI scores were recalculated excluding extreme outliers, all significant effects were retained.
